# LipoxinA4 as a Potential Prognostic Marker of COVID-19

**DOI:** 10.1155/2022/8527305

**Published:** 2022-07-05

**Authors:** Farzaneh Jamali, Bita Shahrami, Amirmahdi Mojtahedzadeh, Farhad Najmeddin, Amir Ahmad Arabzadeh, Azar Hadadi, Mohammad Sharifzadeh, Mojtaba Mojtahedzadeh

**Affiliations:** ^1^Department of Clinical Pharmacy, Faculty of Pharmacy, Tehran University of Medical Sciences, Tehran, Iran; ^2^Semmelweis University, Faculty of Medicine, Budapest, Hungary; ^3^Department of Surgery, Faculty of Medicine, Ardabil University of Medical Sciences, Ardabil, Iran; ^4^Department of Infectious Disease, Faculty of Medicine, Tehran University of Medical Sciences, Tehran, Iran; ^5^Department of Toxicology, Faculty of Pharmacy, Tehran University of Medical Sciences, Tehran, Iran

## Abstract

This pilot study aimed to determine early changes of LXA_4_ levels among the hospitalized patients confirmed as COVID-19 cases following the clinical management and its correlation with commonly used inflammatory markers, including erythrocyte sedimentation rate (ESR), c-reactive protein (CRP), and ferritin. Thirty-one adult hospitalized patients infected with the non-severe COVID-19 were included. LXA_4_ levels were measured at the baseline and 48-72 hours after hospitalization. Accordingly, ESR and CRP levels were collected on the first day of hospitalization. Moreover, the maximum serum ferritin levels were determined during the five days. LXA_4_ levels significantly increased at 48-72 hours compared to the baseline. ESR, CRP, and ferritin levels were positively correlated with the increased LXA4. In contrast, aging was shown to negatively correlate with the increased LXA_4_ levels. LXA_4_ may be known as a valuable marker to assess the treatment response among non-elderly patients with non-severe COVID-19. Furthermore, LXA_4_ could be considered as a potential treatment option under inflammatory conditions. Further studies are necessary to clarify LXA_4_ role in COVID-19 pathogenesis, as well as the balance between such pro-resolving mediators and inflammatory parameters.

## 1. Introduction

Inflammation is an active process associated with the anti-inflammatory mediators' production [1]. These mediators, called specialized pro-resolving mediators (SPMs), are a group of bioactive lipids (BALs). Lipoxins (LXs), are a member of SPMs, involved in the active phase of the resolution, which is thought to control inflammation, promote healing, and limit tissue damage [2]. It is believed that a complex correlation exists between inflammatory and non-inflammatory mediators in several inflammatory and infectious diseases, including coronavirus disease 2019 (COVID-19) [3–5].

## 2. Materials and Methods

An observational pilot study was performed on adult patients hospitalized due to non-severe COVID-19. Serum LXA_4_ levels were measured once at the time of admission and once 48-72 hours later by human ELISA kit in every patient. The assay range of the ELISA kit was from 75 to 2400 ng/L, and the sensitivity was measured as 9.5 ng/L. Therefore, LXA_4_ levels below the detection value (<9.5 ng/L) were considered as 9.5 ng/L for the statistical analysis. Baseline erythrocyte sedimentation rate **(**ESR) and c-reactive protein (CRP) levels and the maximum ferritin level during the five days of hospitalization were also measured.

## 3. Results

The data obtained from a total of 31 patients were analyzed. The mean (±SD) age of the patients, was 61.9 ± 17 years old.

**Figure 1:**
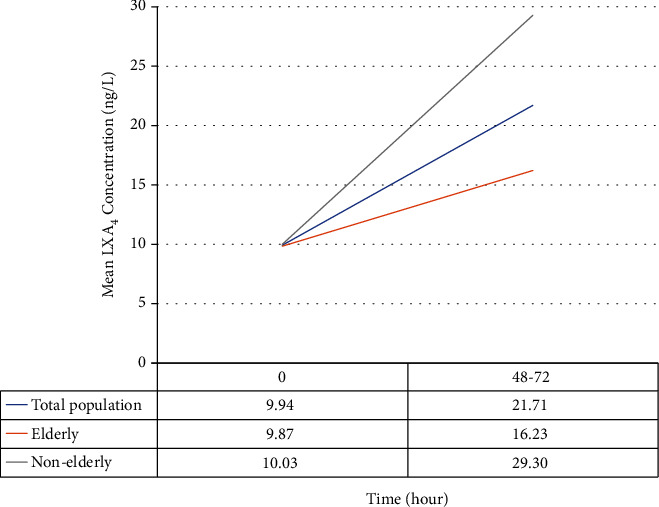
LXA_4_ level changes by age.

Changes of LXA_4_ levels by age are shown in Figure 1. LXA_4_ concentrations significantly increased after early interventions in the hospital (*P* < 0.05). In addition, there was an inverse correlation between age and changes in LXA_4_, indicating that LXA_4_ levels less increased with aging (*R* = -0.375; *P* = 0.037).

The mean±SD baseline concentrations of ESR, and CRP as well as the maximum concentration of ferritin were 55.7 ± 33.7mm/h, 74.7 ± 57.3mg/L, and 568.7 ± 530.0ng/mL, respectively. The results show that the patients with higher serum ESR and CRP levels at the time of admission also had a greater increase in LXA_4_ concentration (*R* = 0.535, 0.499; *P* = 0.005, 0.007, respectively). The positive correlation between the maximum ferritin levels and the LXA_4_ changes was statistically significant as well (*R* = 0.398; *P* = 0.043).

## 4. Discussion

LXs are anti-inflammatory mediators that increase at early stages of resolution [6]. Despite conducting extensive research on the pro-inflammatory mediators, the pro-resolving roles in COVID-19 have been poorly studied. This pilot study showed that LXA_4_ levels increased in the hospitalized patients with non-severe COVID-19 following performing the early therapeutic interventions.

Yang et al. in their study [7] showed that LXs and other SPMs have protective effects in pulmonary diseases associated with inflammation. Additional studies [8, 9] have shown a significant correlation between chronic obstructive pulmonary disease (COPD), severe asthma, and low levels of LXA_4_ [10]. SPMs have shown positive effects on treating sepsis, improving survival, and reducing the need for antibiotics as well [11].

There are studies demonstrating that when human cells are exposed to SARS-CoV-2, they secrete large amounts of BALs [12, 13]. Archambault et al. [14] reported that LXA_4_ is detectable in the bronchoalveolar lavage fluid (BALF) of severe COVID-19 patients. In addition, the levels of SPMs and pro-inflammatory lipids were simultaneously high, indicating the coexistence of such mediators in the acute phase of inflammation, while the resolution process was not fully engaged. LXA_4_ was indicated to have a significant correlation with prostaglandin E_2_, prostaglandin D_2_, and thromboxane B_4_, but it had no significant correlation with clinical parameters and aging. In the present study, in contrast, the changes of LXA_4_ were observed to be correlated with ESR, CRP, and ferritin indicating that the patients with higher inflammatory states secreted more LXA_4_. Another finding of this study was the significant inverse correlation between aging and changes in LXA_4_ concentration. The effects of aging factors on COVID-19 have been previously investigated [15–17]. The baseline inflammation predisposes the elderly to hyper-inflammatory response and impaired resolution [18] which is consistent with our results.

The present study suggests that along with the inflammatory response to pathogens, active resolution, and the timely conversion to resolution phase are of great importance in having an effective fight on the coronavirus. Therefore, SPMs without any immunosuppressive effect may be potential treatment options under inflammatory conditions. Besides, early changes of LXA_4_ in our study showed that such mediators might be valuable markers for assessing the treatment response compared to common-used inflammatory markers with fewer changes. However, the LXA_4_ use is not recommended in the elderly population because of undetectable low levels.

## 5. Conclusion

LXA_4_ is suggested to be a beneficial biomarker in infectious and inflammatory diseases like COVID-19.

## Data Availability

All data generated or analyzed during this study are included in this published article.
